# Increased biofilm formation ability in *Klebsiella pneumoniae* after short‐term exposure to a simulated microgravity environment

**DOI:** 10.1002/mbo3.370

**Published:** 2016-05-16

**Authors:** Haili Wang, Yanfeng Yan, Dan Rong, Jing Wang, Hongduo Wang, Zizhong Liu, Jiaping Wang, Ruifu Yang, Yanping Han

**Affiliations:** ^1^ State Key Laboratory of Pathogen and BiosecurityBeijing Institute of Microbiology and EpidemiologyBeijing100071China; ^2^ Department of Medical Monitoring and SupportAstronaut Center of ChinaBeijing100094China; ^3^ Animal Husbandry Base Teaching and Research SectionCollege of Animal Science and TechnologyHebei North UniversityZhangjiakouHebei075131China

**Keywords:** Biofilm, cellulose, *Klebsiella pneumonia*, simulated microgravity, type 3 fimbriae.

## Abstract

Biofilm formation is closely related to the pathogenetic processes of *Klebsiella pneumoniae*, which frequently causes infections in immunocompromised individuals. The immune system of astronauts is compromised in spaceflight. Accordingly, *K. pneumoniae*, which used to be isolated from orbiting spacecraft and astronauts, poses potential threats to the health of astronauts and mission security. Microgravity is a key environmental cue during spaceflight. Therefore, determining its effects on bacterial biofilm formation is necessary. In this study, *K. pneumoniae* ATCC BAA‐1705 was exposed to a simulated microgravity (SMG) environment. *K. pneumoniae* grown under SMG formed thicker biofilms compared with those under normal gravity (NG) control after 2 weeks of subculture. Two indicative dyes (i.e., Congo red and calcofluor) specifically binding to cellulose fibers and/or fimbriae were utilized to reconfirm the enhanced biofilm formation ability of *K. pneumoniae* grown under SMG. Further analysis showed that the biofilms formed by SMG‐treated *K. pneumoniae* were susceptible to cellulase digestion. Yeast cells mannose‐resistant agglutination by *K. pneumoniae* type 3 fimbriae was more obvious in the SMG group, which suggests that cellulose production and type 3 fimbriae expression in *K. pneumoniae* were both enhanced under the SMG condition. Transcriptomic analysis showed that 171 genes belonging to 15 functional categories were dysregulated in this organism exposed to the SMG conditions compared with those in the NG group, where the genes responsible for the type 3 fimbriae (*mrkABCDF*) and its regulator (*mrkH*) were upregulated.

## Introduction

Several opportunistic bacterial pathogens have been detected in samples from human space habitats and postflight astronauts (Castro et al. 2004; Novikova 2004). Microgravity is a key environmental factor in spaceflight, where microbes sense and respond to environmental stresses by modulating their gene expressions and altering their physiological and pathogenic processes (Wilson et al. 2007; Crabbe et al. 2011). Research resources in microgravity condition are extremely restricted because of the logistic reasons and safety considerations. To address this problem, high‐aspect ratio rotating‐wall vessels (HARVs) are extensively applied to investigate the physiological characteristics of microbes in a simulated microgravity (SMG) environment (Nickerson et al. 2000; Wilson et al. 2002a,b; Lynch et al. 2004, 2006; Crabbe et al. 2010; Lawal et al. 2010; Castro et al. 2011).

HARVs can create a low‐fluid shear condition after being filled with fluid medium and rotated at a setting speed, because air bubbles are completely removed and turbulent flow is minimized. The cells weight in the HARVs of the SMG group is offset with hydrodynamic forces (e.g., centrifugal, Coriolis, and shear components) and constantly suspended in a low‐fluid shear condition, which partially mimics the true microgravity environment. Several previous studies have adopted low‐shear modeled microgravity (LSMMG) to refer to the SMG condition in HARVs. Anaerobic culture is also avoided by oxygen diffusion through a gas‐permeable membrane in the back of HARVs during growth (Schwarz et al. 1992; Hammond and Hammond 2001).

*Klebsiella pneumoniae* is a ubiquitous opportunistic pathogen, commonly found in both clinical and nonclinical settings, and is associated with hospital‐acquired urinary, respiratory tract infections, bacteremia, and surgical wound infections, especially in immunocompromised individuals (Podschun and Ullmann 1998). This organism has been isolated from the equipment of on‐board spacecraft and from astronauts (Taylor 1974; Novikova 2004), and poses potential threats to the health of astronauts because their immune system is compromised in spaceflight (Taylor et al. 1996; Gueguinou et al. 2009). The biofilms alter the adaptation ability of bacterial cells to stressful environments, including antibiotic exposure and host immune responses, and might account for antibiotic treatment failure in chronic infection (Walters et al. 2003; Jefferson et al. 2005). The SMG effects on *K. pneumoniae* biofilm formation were examined in this study by using HARVs to continuously culture this organism under SMG and NG conditions.

## Materials and Methods

### Bacterial strains and growth conditions

**Figure 1:**
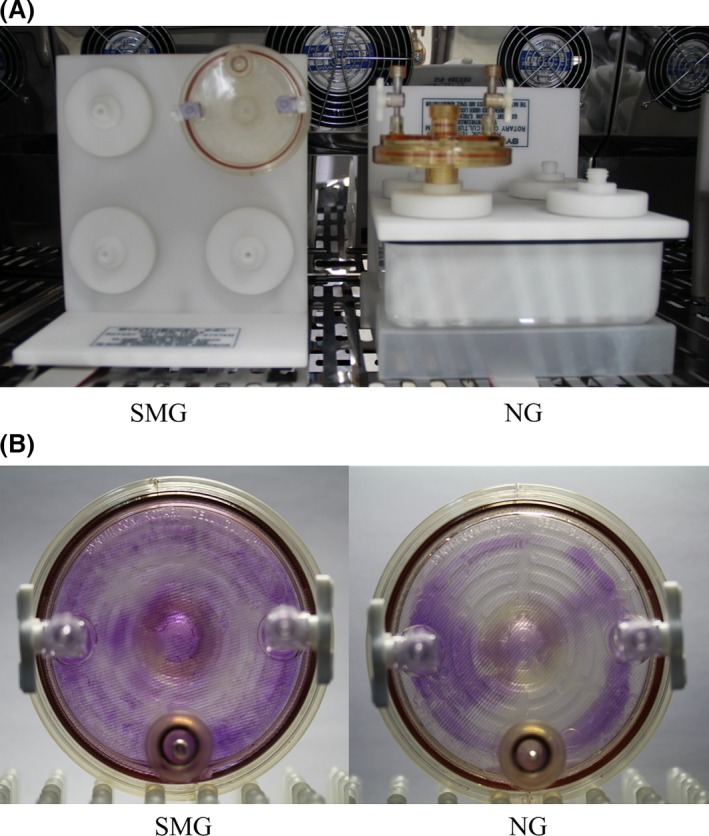
HARV bioreactors in the experimental setup. The bacterial cells in the HARV bioreactor are grown under the simulated microgravity (SMG) condition with its axis of rotation perpendicular to gravity or grown under the NG condition with its axis of rotation vertical to gravity when the medium is filled and the bubbles are removed (A). Both SMG and NG HARV bioreactors are stained with 0.1% crystal violet after 2‐week cultivation (B).

The carbapenem‐resistant *K. pneumoniae* strain ATCC BAA‐1705 was clinically isolated from the urine of a male patient and utilized throughout this study. The bacterial strain was aerobically grown at 37°C in lysogeny broth (LB) or agar unless indicated otherwise. The test (SMG) and the control (NG) settings were established by growing bacterial cells for continuous cultivation in HARV bioreactors (Synthecon, Inc., Houston, Tex, USA). Figure 1A shows the SMG cultivation achieved by rotating the bioreactor with its axis perpendicular to gravity. NG cultivation was achieved with its axis parallel to gravity (Nickerson et al. 2000). Overnight cultures grown at 37°C with shaking at 200 rpm were inoculated at a dilution of 1:200 in the HARV bioreactors. Each bioreactor was completely filled with ~58 mL fresh LB medium. Air bubbles were carefully removed. After 24 h of incubation at 37°C in HARVs with a rotation of 25 rpm, both the SMG and NG bacterial cultures were diluted into new HARVs completely filled with the LB medium and then incubated at 37°C and 25 rpm for another 24 h. Experimental manipulations of bacterial inoculation in the HARV bioreactors were successively performed for 2 weeks. Bacterial cell numbers in the SMG and NG groups were counted through serial dilution in phosphate‐buffered saline (PBS) and plating on LB agar. The resulting cultures were subjected to the following assays.

### Crystal violet staining

Crystal violet staining was performed to evaluate the biofilm formation ability in *K. pneumoniae* after 2 weeks cultivation under SMG and NG conditions. The cultures in the HARV bioreactors were removed. The bioreactors were washed gently with deionized water and then stained with 0.1% crystal violet dye for 15 min at room temperature. Both the test and control cultures were separately diluted (1:100) in 5 mL LB medium in glass tubes and grown at 37°C and 200 rpm for 24 h. The planktonic bacteria were removed. Subsequently, each tube was washed three times with deionized water. The glass tubes were then stained with 0.1% crystal violet dye for 15 min at room temperature.

The biofilm formation in the SMG and NG groups was quantified by separately diluting both the test and control cultures (1:100) on a 24‐well plate. Each well contained 1 mL LB medium. The planktonic bacteria were removed after the 24‐well plate was incubated at 37°C and 200 rpm for 24 h. Each well was washed three times with deionized water. The 24‐well plate was then incubated at 80°C for 15 min to fix the biofilms. The adherent bacterial cells were stained with 0.1% crystal violet dye for 15 min and subsequently rinsed with deionized water. Bound crystal violet was solubilized with 2 mL dimethyl sulfoxide and quantified by measuring the optical density (OD) values at 570 nm. The results are presented as mean ± SD for three biological replicates.

### Congo red‐based colony morphology

Ten microliters of 2‐week cultures under both NG and SMG conditions were spotted onto an LB agar plate containing 25 *μ*g/mL Congo red. The spotted cultures were incubated at 37°C. The images of the spotted cultures were captured after 72 h.

### Calcofluor staining

Five milliliters of *K. pneumoniae* cultures under both NG and SMG conditions were harvested and resuspended in 5 mL of PBS with 200 *μ*g/mL calcofluor. The cells were recovered by centrifugation after 10 min of incubation at room temperature. The cells were then rinsed and resuspended with 5 mL PBS. Afterward, 10 *μ*L of the resuspended mixture was dropped onto plastic plates. The fluorescent colony was visualized by exposure to UV light and imaged by using a UV light imaging system (Gel Doc^TM^ XR+, Bio‐Rad). For fluorescence quantification, 100 *μ*L bacterial cell suspension was added to 96‐well black cell culture plates (Corning^®^ Costar^®^). The fluorescence values were then measured with a spectrophotometer (SpectraMax M2, Molecular Devices) with 366 nm excitation and 525 nm emission. The results are indicated as mean ± SD calculated from three replicate samples.

### Cellulase digestion of biofilms

The biofilm formation of the SMG and NG cultures was measured in 24‐well plate as described in the “Crystal violet staining” section. Each well was rinsed with deionized water after the planktonic bacteria were removed. Two milliliters of 50 mmol/L citrate buffer (pH 4.6) with 0.1% cellulase (Sigma Chemical Co., St. Louis, Mo, USA) were added to the wells, which were then incubated at 45°C for 48 h (Huertas, Zarate et al. 2014). The biofilms were stained with crystal violet and quantified through spectrometry.

### Yeast cell agglutination assays

The PBS‐resuspended cultures in both SMG and NG groups were serially and doubly diluted in 24‐well plates to identify the yeast cell agglutination titers. Briefly, 100 *μ*L of the PBS‐resuspended bacterial cells of each dilution degree in both the SMG and NG groups was applied to a polystyrene plate and mixed with 50 *μ*L of 1% yeast cells (Sigma Aldrich) and 50 *μ*L of 5% mannose (Sigma Aldrich). The polystyrene plate was rotated until agglutination was visible (Stahlhut, Struve et al. 2012). The logarithm means of the agglutination titers in both the SMG and NG groups were obtained from at least three experiments.

### RNA‐seq‐based transcriptional analysis

The total RNAs of the bacterial cells incubated in the SMG and NG bioreactors were extracted with the PureLink^™^ RNA Mini Kit (Ambion, USA) according to the manufacturer's instructions. RNA quality was checked through agarose gel electrophoresis. The RNAs were then quantified with a spectrophotometer. The RNA samples were subsequently subjected to cDNA library construction and deep sequencing. The fragments per kilobase of transcript per million fragments mapped values were calculated to determine the expression levels of transcripts. The fold changes in the transcript levels between the SMG and NG groups were also calculated. A twofold change was defined as the arbitrary threshold of the differentially regulated genes.

### Quantitative real‐time PCR

cDNA was synthesized by 7 *μ*g of the total RNAs and 3 *μ*g of random hexamer primers with the Superscript III reverse transcriptase (Invitrogen) for quantitative real‐time PCR (qRT‐PCR). All primer pairs were designed to produce amplicons with expected sizes of 100–200 nt using *K. pneumoniae* ATCC BAA‐1705 genomic DNA as the PCR template (Table 1). qRT‐PCR was performed in duplicate for each RNA sample using the LightCycler system (Roche, Switzerland) with cDNA as a template. The relative fold change of the target genes in the test and control RNA samples was determined. The *16S rRNA* gene was used as an internal reference.

**Table 1:** Primers for the target genes used in qRT‐PCR

Gene name	Primers (forward/reverse, 5′–3′)
*bcsA*	GCTTGGCTGTCTGTGGG/GCGATGCGGATTTATGTG
*mrkA*	TTCTTCTCTCTGCAGCAATG/TACCGGAGACAGGTAAACGT
*mrkB*	ACCCGCTTTATTTATCCAGG/AAACGGGGTGGTAATGGTAT
*mrkC*	TGTGCTGCTTTCCGCCATTT/CGCCCTTTCCACTCGTCGTT
*mrkD*	GCGTCTCTCATCGCCAACGG/CACGATCTTCGCCGCAAAGC
*mrkF*	CCTCGGCGTGGGGTTTTGAG/GACTTCCGCCAGGCTGACCG
*mrkH*	TGGACTTTGCCGAGTT/ACCGCTATTGTCATGTTT
16S rRNA	GAGCGGCGGACGGGTGAGTA/GGGCACATCTGATGGCATGA

### Statistical analysis

All the quantitative experiments were independently performed at least in triplicate. The data were analyzed with SPSS 20.0 (SPSS, Chicago, IL, USA), and Student's *t*‐test was utilized to determine the statistical significance (*P *<* *0.05) between any two groups.

## Results

### Enhanced effect of SMG on *K. pneumoniae* biofilm formation

**Figure 2:**
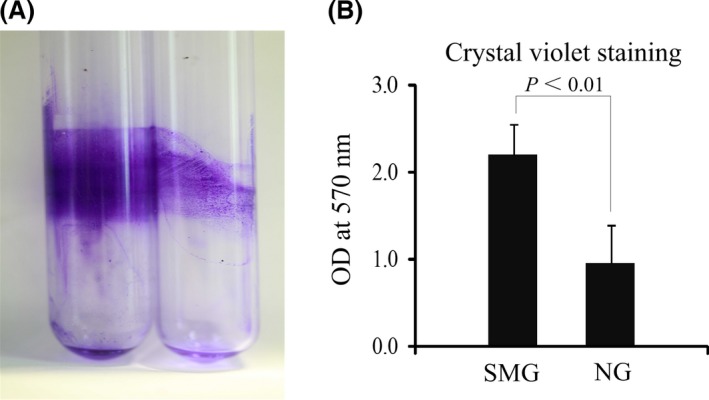
Quantification of biofilm formation by *K. pneumoniae* grown under SMG and NG conditions. The biofilms formed on the glass tubes are monitored by crystal violet staining (A) and quantified by measuring the OD_570_ values (B). SMG, simulated microgravity.

The biofilm formation phenotypes of both the SMG and NG cultures were analyzed after 2‐week continuous cultivation by observing the pellicles that formed and adhered to the gas‐permeable membrane at the back of the bioreactors and the glass tubes at the air–liquid interface. OD_570_ was also measured through crystal violet staining. The pellicles formed by *K. pneumoniae* grown under the SMG condition were thicker than those grown under the NG condition (Figs. 1B and 2A). The adhered pellicles were further quantified by the OD_570_ values of crystal violet staining. More than a twofold increase in the adhered dye was observed in *K. pneumoniae* under the SMG condition relative to the NG condition (Fig. 2B). The cell numbers in the SMG and NG groups were also counted by dilution in PBS and plating on LB agar for three times (~2.1 × 10^9^ CFU/mL and 3.2 × 10^9^ CFU/mL in the SMG and NG groups, respectively). This process confirmed that the thicker biofilm formation in the SMG group was not caused by more bacterial cell inoculation in the SMG group.

### Increased Congo red binding to the colony and yeast cell agglutination formed by *K. pneumoniae* grown under SMG

**Figure 3:**
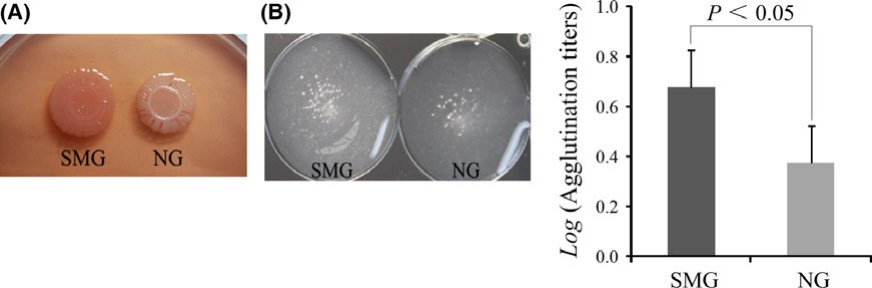
Colony morphology observation and type 3 fimbriae expression analysis. Colony morphology of the simulated microgravity (SMG)‐ and NG‐treated *K. pneumoniae* on Congo red agar (A). *Klebsiella pneumoniae* type 3 fimbriae can agglutinate yeast cells in a mannose‐resistant manner. Yeast cell agglutination (induced by undiluted bacterial cells) is observed on polystyrene plates for the SMG and NG groups. The agglutination titers of *K. pneumoniae* type 3 fimbriae in the SMG and NG groups are also compared by their logarithm means (B).

The major biofilm components were identified by spotting the bacterial culture on Congo red agar plates. The *K. pneumoniae* in the SMG group resulted in an RDAR (red, dry, and rough) colony, whereas the NG group remained white (Fig. 3A). The RDAR morphology is often associated with cellulose and fimbriae production characterized in *Salmonella enteric* serovar Typhimurium and *Escherichia coli* (Zogaj, Nimtz et al. 2001). The results of the different binding abilities of Congo red dye indicated that SMG might promote cellulose and fimbriae expression, which results in increased biofilm formation in *K. pneumoniae*.

The agglutination assays also showed that yeast cell agglutination by *K. pneumoniae* type 3 fimbriae in a mannose‐resistant manner was more obvious in the SMG group than in the NG group. The geometric mean of the agglutination titers in the SMG group was 1:4.8, whereas that in the in the NG group was 1:1.7. And the logarithm mean of the agglutination titers in the SMG higher than that in the NG groups (Fig. 3B). This result indicates that a higher type 3 fimbriae expression exists in the SMG‐treated *K. pneumoniae*.

### Cellulose as a major component of the biofilms formed by *K. pneumoniae* grown under SMG

**Figure 4:**
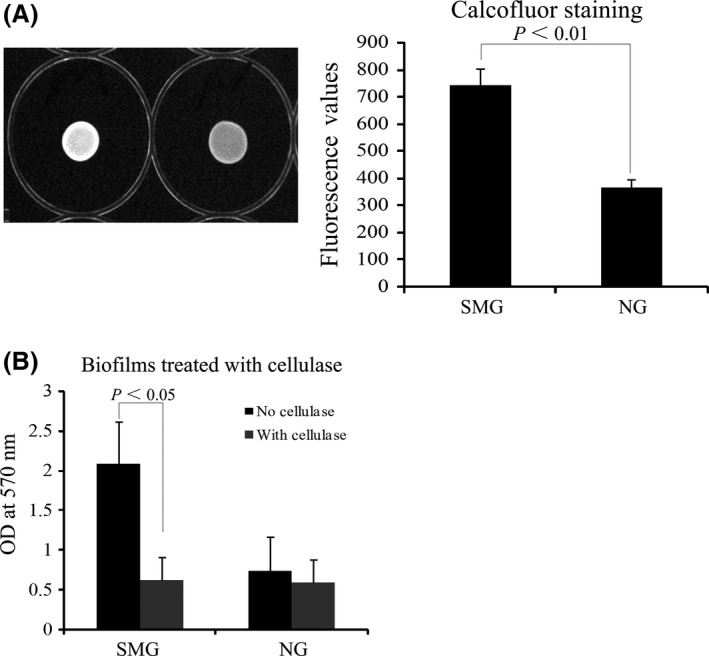
Cellulose production assay for *K. pneumoniae* cultured under SMG and NG conditions. Cellulose production is monitored by calcofluor staining (A). The biofilms formed in the wells of the 24‐well plate are quantified by crystal violet staining after digestion with cellulase (B). SMG, simulated microgravity.

Cellulose participates in the biofilm formation process in many bacteria (Romling 2002). The fluorescent dye, calcofluor, specifically binds to cellulose but not to curli fimbriae. The dye binds *β*‐1, 4 glucose cellulose linkages and causes a fluorescence strain phenotype under a long‐wavelength UV light source. Hence, calcofluor dye is widely used to analyze cellulose production in bacteria (Zogaj, Bokranz et al. 2003). The calcofluor fluorescence phenotype was detected to access cellulose production in *K. pneumoniae* under SMG and NG conditions. The fluorescence intensity of the SMG‐treated *K. pneumoniae* in the calcofluor agar was higher than that of the NG‐treated group. Quantitative results show that the fluorescence values at 525 nm of the SMG cultures are approximately twice those of the NG cultures (Fig. 4A). This result suggests that cellulose production increased after exposure to the SMG condition.

The existence of cellulose in the biofilms was further confirmed by adding cellulase to digest the cellulose within the biofilms. A sharp decrease in the OD_570_ values was observed in the SMG group upon cellulase treatment, whereas no significant difference was found in the NG group under the same condition (Fig. 4B). Cellulose might be an important component of thick biofilms in SMG‐treated *K. pneumoniae*.

### Increased transcriptional level of genes for type 3 fimbriae in SMG‐treated *K. pneumoniae*

RNA‐seq was utilized to monitor differences in the transcriptomic mRNA profile at a global scale between the SMG‐ and NG‐treated *K. pneumoniae*. A total of 171 genes were differentially expressed in the SMG group relative to the NG group (i.e., 89 upregulated genes and 82 downregulated genes) (see Data S1). The differentially regulated genes were then classified based on the KEGG pathway database. A total of 88 genes were categorized to 15 biochemical or signal transduction pathways. Approximately 64% (i.e., 56 genes) of the dysregulated genes belonged to five “metabolic pathway” categories (i.e., amino acid, carbohydrate, and energy metabolisms) (Table 2). A total of 14 dysregulated genes involved in mRNA translation were upregulated in *K. pneumoniae* grown under the SMG condition.

**Table 2:** KEGG pathways of dysregulated genes in *K. pneumoniae* grown under SMG

No.	Pathway	Upregulated genes		Downregulated genes	
		No.	Gene ID (KPBAA1705_)	No.	Gene ID (KPBAA1705_)
1	Amino acid metabolism	5	06833, 14946, 14961, 17616, 25221	9	03301, 10183, 13245, 19907, 19912, 20820, 21421, 23663, 23678
2	Carbohydrate metabolism	2	16206, 25221	10	05906, 06221, 10902, 14095, 20210, 20820, 23023, 23323, 23663, 26174
3	Cell growth and death	0	** **	1	25071
4	Energy metabolism	7	05591, 05596, 13845, 13850, 13855, 13860, 25221	1	10183
5	Glycan biosynthesis and metabolism	3	14946, 14961, 14966	2	14095, 23023
6	Virulence	3	02841, 02891, 13830	0	** **
7	Lipid metabolism	2	14395, 25221	4	14095, 20820, 23023, 23663
8	Membrane transport	4	02836, 13820, 13830, 15096	4	02701, 05936, 24409, 26184
9	Metabolism of cofactors and vitamins	1	02401	** **	** **
10	Metabolism of terpenoids and polyketides	2	19092, 25221	1	23663
11	Nucleotide metabolism	1	00965	0	** **
12	Replication and repair	4	02586, 19637, 27259, 27454	0	** **
13	Signal transduction	2	13550, 13830	1	23323
14	Translation	14	21446, 26464, 26469, 26474, 26479, 26484, 26489,26494, 26499, 26504, 26509, 26514, 26554, 26559	0	** **
15	Xenobiotics biodegradation and metabolism	1	25221	3	03301, 23323, 23663

**Figure 5:**
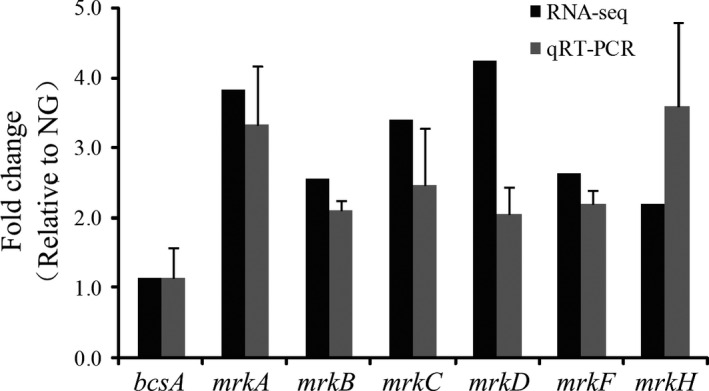
Expression validation of the biofilm‐associated genes by RNA‐seq (black) and qRT‐PCR (gray). Relative expression is indicated as fold changes ± standard derivation between the SMG‐ and NG‐treated *K. pneumoniae*. SMG, simulated microgravity.

The biofilm formation ability of *K. pneumoniae* is determined by the intracellular levels of capsule, exopolysaccharides, and cell surface fimbriae (Jagnow and Clegg 2003; Schroll, Barken et al. 2010; Huertas, Zarate et al. 2014). Therefore, the expressions of the cellulose synthase‐encoding gene *bcsA* and type 3 fimbriae‐encoding genes *mrkABCDF* and its regulatory gene *mrkH* were selected for further analysis through RNA‐seq and qRT‐PCR. As expected, the *mrkABCDF* and *mrkH* gene expressions were upregulated in the SMG‐treated *K. pneumoniae*. However, the *bcsA* gene expression was unchanged at the transcriptional level (Fig. 5).

## Discussion

The bacterial cells in biofilms are embedded in extracellular polymeric substances (EPS) mostly produced by themselves, including exopolysaccharides, proteins, extracellular DNA, surfactants, lipids, and water (Flemming and Wingender 2010). Cellulose, an exopolysaccharide composed of *β*‐1, 4 glycosidic bond‐linked glucose units, has been identified as a significant component of EPS associated with biofilm formation in various bacteria (Zogaj, Nimtz et al. 2001; Spiers, Sm. et al. 2003). Extracellular structures (pili or fimbriae) in EPS are also required for biofilm formation in *Enterobacteriaceae* (Pratt and Kolter 1998). The altered biofilms in bacteria often correlate with the fluctuant binding ability of the Congo red dye and the fluorescence dye calcofluor. These two indicative dyes can specifically bind to cellulose and/or fimbriae. Thus, both dyes were utilized in this study to assess biofilm formation and ascertain the major components of thick biofilms formed by the SMG‐treated *K. pneumoniae*.

Cellulose biosynthesis in *K. pneumoniae* is silent under laboratory conditions (Zogaj, Bokranz et al. 2003). The present study showed that cellulose production was enhanced and mainly responsible for biofilm formation in *K. pneumoniae* grown under the SMG condition. However, no significant difference in the cellulose synthase‐encoding *bcsA* transcript abundance of *K. pneumoniae* was observed between the SMG and NG groups. Given the previous finding that cellulose synthesis is positively regulated by c‐di‐GMP via the allosteric effect on BcsA, the high cellulose production and the indistinctive value of *bcsA* transcript abundance in the SMG group may be attributed to the fact that *bcsA* expression is mainly regulated at the posttranslational level in the SMG condition (Morgan, Strumillo et al. 2013).

In *K. pneumoniae*, the type 3 fimbriae mediate the biofilm formation on biotic and abiotic surfaces (Murphy and Clegg 2012). Type 3 fimbriae are encoded by the *mrkABCDF* operon, *mrkA*, which encodes the major fimbria subunit; *mrkB* and *mrkC*, which encode a chaperone–usher system; *mrkD*, which encodes the fimbrial tip ahesin; and *mrkF*, which encodes an unknown function protein. The type 3 fimbriae expression is also regulated by the c‐di‐GMP molecule. The *mrk* gene cluster expression is activated at the transcriptional level via the c‐di‐GMP‐dependent regulatory effect on MrkH (Wilksch, Yang et al. 2011). The yeast cell agglutination assays and the transcriptomic analysis in this study indicated that the expression of *K. pneumoniae* type 3 fimbriae was upregulated in the SMG‐treated group.

Previous reports have shown that the expression of numerous genes is affected for bacterial cells in simulated microgravity (Wilson et al. 2002b; Crabbe et al. 2010). The transcriptomic analysis in this study showed that 171 genes belonging to 15 functional categories were dysregulated in *K. pneumoniae* of the SMG group. Among these dysregulated genes, 45 gene products were hypothetical proteins; the others were not closely related to *K. pneumoniae* biofilm formation, except for cellulose and the type 3 fimbriae‐encoding genes. The expression of type 1 fimbriae (encoding by the *fimBEAICDFGHK* gene cluster) subunit‐encoding gene *fimA* was also significantly upregulated in *K. pneumoniae* of the SMG group. But no other correlated gene upregulation or expression was identified in both groups, which indicates that type 1 fimbriae were not correctly expressed in the *K. pneumoniae* cultured in the HARVs.

Biofilm formation in bacteria was first investigated under microgravity in 2001 (McLean, Cassanto et al. 2001). Short‐term (24 h) exposure to microgravity affects the biofilm production in *E. coli* and *Pseudomonas aeruginosa* (Lynch et al. 2006; Crabbe, De Boever et al. 2008). Li et al. cultured *K. pneumoniae* ATCC BAA‐2146 in a semisolid medium for 15 days in spaceflight. Their results showed an enhanced *K. pneumoniae* biofilm formation ability after cultivation, which was consistent with our results obtained from culturing *K. pneumoniae* in the SMG environment (Jia Li 2014). However, the *K. pneumoniae* cultured in the SMG environment in Li et al.'s study showed a similar biofilm formation capacity with the strain cultured on the ground. Moreover, no gene closely related to biofilm formation was upregulated in spaceflight or SMG condition. The differences in bacteria strains and culture methods (culture medium and microgravity analogs) selected in the studies may be the cause of the different results.

Biofilm communities control the diffusion of nutrition components and shield bacteria from various environmental stress, which may cause the bacteria to colonize inside their hosts and induce infections (Donlan and J William 2002; López, Vlamakis et al. 2010). One of the main habitats of *K. pneumoniae* is the mucosal surfaces of mammals, which includes intestinal, respiratory, and urogenital mucosa. This organism encounters a low‐fluid shear environment in these habitats, which is achieved by the brush border microvilli of epithelial cells (Nickerson, Ott et al. 2004). Epidemiological studies have shown that the usual precondition for *K. pneumoniae* to induce nosocomial infections is its colonization of a patient's gastrointestinal (GI) tract (Mircˇeva, Žigon et al. 1980; De Champs, Sauvant et al. 1989). Thus, the microenvironment in the GI tract may provide a proper niche for *K. pneumoniae* colonization and survival. We believe that to a certain extent, several similarities exist between SMG environments and the mucosal surfaces of mammals. Therefore, the enhanced biofilm formation in the SMG condition might not only show the altered adaptation of *K. pneumoniae* to this special environment, but also provide insights into the mechanisms of *K. pneumoniae* colonization of the human mucosa.

In this study, HARVs are completely filled with LB medium, so low‐fluid shear condition was achieved in both the SMG and NG groups. Although low‐fluid shear condition may be important for *K. pneumoniae* biofilm formation, it is not the main cause of *K. pneumoniae* biofilm formation differences between the SMG and NG groups. Unlike bacterial cell sedimentation in the NG group, cells in the SMG group are constantly suspended by hydrodynamic forces during cultivation, which partially mimics the true microgravity environment and may benefit biofilm formation in *K. pneumoniae*.

HARVs are designed to have a large gas‐permeable membrane and a small thickness value, which guarantee oxygen permeability into the medium. Previous studies on *E. coli* have shown that sufficient oxygen is present for bacterial growth throughout HARVs (Lynch et al. 2006). However, in our study, *K. pneumoniae* biofilm formation on the membrane reduced the oxygen permeability and an oxygen concentration gradient might form in HARVs during the later stage of 24‐h cultivation, which might also be a factor that influenced the growth of *K. pneumoniae*.

Long‐term spaceflight missions of astronauts on space stations require urgent monitoring of the potential changes in bacterial physiology and virulence caused by exposure to prolonged microgravity. Given the safety and constraints of equipment and spaceflight time, the development of earth‐based microgravity analogs to conduct experiments on microbes is necessary. A detailed investigation of the SMG‐regulated genes in this bacterium is currently being undertaken in our laboratory. This investigation would deepen our understanding of the SMG effects on *Klebsiella* physiology and pathogenicity.

## Conflict of Interest

None declared.

## Supporting information


**Data S1.** The dysregulated genes of *K. pneumoniae* cultured in SMG environment.
